# DNA Methylation and Detoxification in the Earthworm *Lumbricus terrestris* Exposed to Cadmium and the DNA Demethylation Agent 5-aza-2′-deoxycytidine

**DOI:** 10.3390/toxics10020100

**Published:** 2022-02-21

**Authors:** Gerhard P. Aigner, Pamela Nenning, Birgit Fiechtner, Maja Šrut, Martina Höckner

**Affiliations:** Department of Zoology, Center for Molecular Biosciences Innsbruck, University of Innsbruck, Technikerstraße 25, 6020 Innsbruck, Austria; gerhard.p.aigner@uibk.ac.at (G.P.A.); pamela.nenning@student.uibk.ac.at (P.N.); birgit.fiechtner@uibk.ac.at (B.F.); Maja.Srut@uibk.ac.at (M.Š.)

**Keywords:** DNA methylation, earthworms, cadmium, epigenetics, metallothionein, DNMT, TET, demethylation, 5-aza-2′-deoxycytidine, decitabine

## Abstract

Earthworms are well-established model organisms for testing the effects of heavy metal pollution. How DNA methylation affects cadmium (Cd) detoxification processes such as the expression of metallothionein 2 (MT2), however, is largely unknown. We therefore exposed *Lumbricus terrestris* to 200 mg concentrations of Cd and 5-aza-2′-deoxycytidine (Aza), a demethylating agent, and sampled tissue and coelomocytes, cells of the innate immune system, for 48 h. MT2 transcription significantly increased in the Cd- and Cd-Aza-treated groups. In tissue samples, a significant decrease in MT2 in the Aza-treated group was detected, showing that Aza treatment inhibits basal MT2 gene activity but has no effect on Cd-induced MT2 levels. Although Cd repressed the gene expression of DNA-(cytosine-5)-methyltransferase-1 (DNMT1), which is responsible for maintaining DNA methylation, DNMT activity was unchanged, meaning that methylation maintenance was not affected in coelomocytes. The treatment did not influence DNMT3, which mediates de novo methylation, TET gene expression, which orchestrates demethylation, and global levels of hydroxymethylcytosine (5hmC), a product of the demethylation process. Taken together, this study indicates that Aza inhibits basal gene activity, in contrast to Cd-induced MT2 gene expression, but does not affect global DNA methylation. We therefore conclude that Cd detoxification based on the induction of MT2 does not relate to DNA methylation changes.

## 1. Introduction

Earthworms, as ecosystem engineers, are important organisms for soil formation because their activities have a tremendous impact on organic matter decomposition, nutrient cycles, bioturbation, aggregate formation, etc. [1]. Over the last few centuries, human activities have caused an accumulation of heavy metals in the environment [2]. The heavy metal cadmium (Cd) is one of the main soil pollutants declared as a carcinogenic substance by the WHO [3]. Thus, a Cd-polluted environment threatens soil life, including earthworms. Due to the chemical and physical similarities of Cd to the trace elements zinc (Zn) and calcium (Ca), substitutions of these elements lead to toxic effects [4]. Metallothioneins (MTs) are cysteine-rich proteins capable of binding Cd [5]. Various ecotoxicological studies in plants, vertebrates and invertebrates have been performed explaining the toxic effects of Cd [6,7,8,9,10]. In the last few decades, increased research effort was put into deciphering epigenetic alterations caused by environmental stressors such as Cd. An altered epigenome can, in turn, play a role in adaption to environmental changes by influencing the activity of beneficial genes [11,12,13]. In the most modern definition, epigenetics is deemed the study of changes in gene functions that are heritable at least from one generation of cells to the next and do not entail a change in DNA sequence [14]. The environment influences epigenetic markers; these modifications can last for a long time and can even be measured across generations [15]. Therefore, epigenetic parameters were suggested as biomarkers for reflecting recent and past pollution burdens [16,17,18].

DNA methylation is the first and best-described epigenetic mechanism and refers to a methyl group added to the fifth carbon of the DNA base cytosine (5mC) and most often takes place at C-G dinucleotide pairs (CpG) [19,20,21]. CpG is methylated by DNA (cytosine-5)-methyltransferase 1 (DNMT1) or 3 (DNMT3), which catalyze the reaction that forms 5mC by transferring a methyl group from S-adenosyl methionine (SAM) to the unmodified cytosine base [22]. DNA is methylated by DNMT3 de novo [23]. DNMT1 passes the methylation pattern on from one generation of cells to the next, which thus is often referred to as the DNA methylation maintenance enzyme [24]. The 5mC is either passively or actively demethylated. Passive demethylation occurs when 5mC spontaneously deaminates to thymine [25] or when repressed DNMT1 or co-factor genes/enzymes lead to a replication-dependent loss of methylation patterns [26]. DNA is actively demethylated by a pathway including ten–eleven translocation (TET), thymine-DNA-glycosylase (TDG) and base excision repair (BER). TET enzymes iteratively oxidize 5mC to 5-hydroxymethylcytosine (5hmC), 5hmC to 5-formylcytosine (5fC) and 5fC to 5-carboxylcytosine (5caC) [27]. Methylation-sensitive transcription factors affect the activity of methylated genes [28,29]. Thus, unfavorable DNA methylation patterning can lead to various diseases, such as cancer [30]. Demethylation agents such as 5-aza-2′-deoxycitidine (Aza), also known as decitabine, are used in medicine to treat hypermethylation-associated ailments against acute myeloid leukemia [31,32]. Aza is a chemical analog of cytidine and is also capable of demethylating genes in invertebrates [33,34,35]. The introduction of Aza to a cell leads to several cascades of chemical reactions that form Aza-triphosphate (Aza-TP), which is incorporated into DNA during the S-phase of the cell cycle instead of cytosine [31,36,37,38]. During DNA replication, hemi-methylated sites normally become methylated by DNMT1, but the carbon at the fifth position is replaced by a nitrogen in Aza-TP. Thus, DNMT1 cannot perform the methyl transfer and remains covalently bonded to the cytosine analog, leading to a passive, replication-dependent loss of DNA methylation [31,39,40].

Organisms show various epigenetic reactions to environmental stressors, which can further differ on the tissue level. For example, in the mussel *Crassostrea gigas* exposed to the herbicide diuron, no significant effects on global methylation levels were observed for whole-tissue DNA, in contrast to the digestive gland [41] and sperm [42]. In the crayfish *Procambarus virginalis*, genes involved in DNA methylation are differentially expressed in certain tissues and specific developmental stages [43]. In *Lumbricus terrestris* coelomocytes, environmentally relevant Cd concentrations induce long-lasting global DNA methylation changes [44]. Coelomocytes are cells of the innate immune system of earthworms and are specialized in stress response, including the expression of MTs [45]. Recently, it was revealed that *L. terrestris* possesses genes responsible for DNA methylation patterning, but those were not responsible for Cd-induced DNA hypermethylation [46]. It was suggested to further study the role of epigenetic biomarkers in ecotoxicology and expand this research field to a variety of organisms and ecosystems [47,48]. However, only few epigenetic studies in ecotoxicology have focused on invertebrates [18].

Therefore, the aim of this study was to check for mechanistic relationships of Cd-induced hypermethylation and MT-mediated Cd detoxification in *L. terrestris* by using high doses of Cd (200 mg Cd/kg soil) and the demethylating agent Aza. More specifically, this study aimed to answer the following questions: (1) Does the short-term exposure to high doses of Cd lead to changes in global DNA methylation patterns, and if so, can this change be related to the induction of MT2?; (2) Do increased levels of Cd affect gene activity of the DNA methylation machinery?; (3) Does Aza treatment decrease global DNA-methylation levels and/or does Aza affect MT2 gene expression in earthworms?

## 2. Materials and Methods

### 2.1. Experimental Setup and Sampling

*Lumbricus terrestris* specimens and soil for acclimation (mixture of humus and peat) were ordered from Proinsects GmbH (Minden, Germany). For acclimation, earthworms were stored for 2 weeks in the soil at 70% soil humidity, 15 °C room temperature, a day-night cycle of 8/16 h and were fed once per week with horse manure. For the experimental setup, one earthworm was placed im a Petri dish filled with either 15 mL phosphate-buffered saline (PBS) (NaCl 137 mM; KCl 2.7 mM; KH_2_PO_4_ 1.76 mM; Na_2_HPO_4_ 10.1 mM), 15 mL PBS spiked with 200 µg/mL CdCl_2_ (Merck, Darmstadt, Germany), 15 mL PBS containing 10 µM Aza (Sigma Aldrich A3656, St. Louis, MO, USA), or 15 mL PBS including both 200 µg/mL CdCl_2_ and 10 µM Aza (CdA). Earthworms were then incubated for 48 h under dark conditions at 15 °C. The numbers of replicates used for specific analyses is given within the figure legends in the results section.

After the treatment, worms were washed with PBS and placed in a new Petri dish containing 7 mL cold guaiacol glyceryl ether phosphate-buffered saline (GGE–PBS) on ice. For non-invasive coelomocyte extrusion, worms were stimulated with a 9 V electric current, resulting in the release of coelomocytes through their dorsal pores [49]. The whole GGE–PBS–cell suspension was transferred into a fresh tube and centrifuged at 4029 rcf at 4 °C for 10 min. The supernatant was discarded, and the cell pellet was resuspended in 1 mL PBS. The cell number was determined using a Countess™ 2 FL Automated Cell Counter (Thermo Fisher Scientific, Waltham, MA, USA). Samples with a cell count above 7 × 10^5^ cells/mL were further processed and centrifuged at 1902 rcf at 4 °C for 4 min. The supernatant was discarded and the pellet was either flash-frozen in liquid nitrogen and stored at ™80 °C until further use or was directly processed for RNA extraction. Directly after coelomocyte extrusion, earthworm tissue samples were taken and stored in 1 mL absolute EtOH (Merck, Darmstadt, Germany) at −20 °C for RNA extraction, or shock-frozen in liquid nitrogen and stored at −80 °C for protein and DNA extraction. The same tissue section was used for the respective analyses.

### 2.2. RNA Extraction and cDNA Synthesis

For RNA extraction, 1 mL TRI REAGENT^®^ (Sigma Aldrich, St. Louis, MO, USA) was added to coelomocyte samples and cells were disrupted by pipetting up and down several times. Tissue samples that were previously frozen were homogenized using six RNase-free glass beads in 1 mL TRI REAGENT^®^, applying the benchtop homogenizer FastPrep-24^TM^ 5G (MP Biomedicals, Santa Ana, CA, USA). Three cycles of 40 s at 6 m/s with a break of 2 min between each cycle were used for tissue disruption. RNA was extracted following the manufacturer’s instructions. DNA was digested using DNAse I (Thermo Fisher Scientific, Waltham, MA, USA), including RiboLock (Thermo Fisher Scientific, Waltham, MA, USA) as an RNAse inhibitor. The quality of extracted RNA was evaluated by gel electrophoresis, assessing rRNA band integrity. The RNA concentration was measured in triplicate, using the Quant-iT^TM^ RiboGreen^TM^ RNA Assay Kit (Thermo Fisher Scientific, Waltham, MA, USA) on the plate reader Victor X4 (Perkin Elmer, Waltham, MA, USA). Subsequently, 450 ng of total RNA was used for reverse transcription using RevertAid^TM^ H Minus Reverse Transcriptase (Thermo Fisher Scientific, Waltham, MA, USA), Random Hexamer Primers (Thermo Fisher Scientific, Waltham, MA, USA), and RiboLock (Thermo Fisher Scientific, Waltham, MA, USA), according to the user manual. To eliminate possible contamination with humic acid, an additional washing step was performed using the RNeasy^®^ MinElute^®^ Cleanup Kit (QIAGEN, Hilden, Germany), according to the manufacturer’s instructions.

### 2.3. Quantitative Real-Time PCR

Absolute quantification using a standard curve prepared from a series of dilutions of known template concentrations and a primer matrix was applied to determine optimal primer concentrations. Copy numbers were measured on a QuantStudio^TM^ 3 Real-Time PCR system (Thermo Fisher Scientific, Waltham, MA, USA) using previously published primers [46]: 5 µL Power SYBR^TM^ Green PCR-Master-Mix (Thermo Fisher Scientific, Waltham, MA USA) was used per sample. DNMT1 and DNMT3 were amplified as follows: 1 µL 10 × BSA, 1 µL forward primer (9 µM), 1 µL reverse primer (9 µM), 1 µL distilled water and 1 µL cDNA. TET was amplified by adding 1 µL 10 × BSA, 1 µL forward primer (3 µM), 1 µL reverse primer (3 µM), and 2 µL cDNA. For MT2, 1 µL 10 × BSA, 1 µL forward primer (9 µM), 1 µL reverse primer (9 µM), and 2 µL cDNA. The following conditions were applied: 50 °C for 2 min; 95 °C for 10 min; 40 repeats of 95 °C for 15 s and 60 °C for 1 min. Three technical replicates were generated for each sample. According to the standard curve, absolute copy numbers per 100 ng RNA were determined. To compare different plates, values were normalized to the threshold. Values that were below the detection range of our standard curve were set to zero.

### 2.4. Protein Extraction and DNMT Activity

The extraction of coelomocyte proteins was performed as previously described for aphids [50]: 100 µL proteinase inhibitor buffer (150 mM NaCl, 50 mM Na_2_HPO_4_, 50 mM NaHPO_4_, pH 7.5), 10 µL PMSF (1 mM) and 10 µL DTT (1 mM) were added to frozen coelomocyte samples and a micro pestle was used to homogenize the cells. Homogenates were centrifuged at 21,130 rcf at 4 °C for 10 min, and the supernatant containing the nuclear fraction was transferred to a clean tube. The protein concentration was measured using a NanoDrop^TM^ 2000 (Thermo Fisher Scientific, Waltham, MA, USA). The protein activity of DNMT was quantified using the colorimetric DNMT Activity Quantification Kit (ab113467, Abcam, Cambridge, UK) by following the user manual for nuclear fractions.

### 2.5. Quantification of 5-Methylcytosine (5mC) and 5-Hydroxymethylcytosine (5hmC)

Genomic DNA methylation levels were determined from the same individuals that were also used for quantitative real-time PCR. Genomic DNA of tissue samples was extracted using the GenElute^TM^ Mammalian Genomic DNA Mini-prep Kit (Sigma-Aldrich, St. Louis, MO, USA) according to the manufacturer’s instructions: 25 mg samples of frozen tissue were homogenized using Lysis Solution T and Proteinase K at 55 °C for 4 h, and DNA was stored at 4 °C. DNA extraction from coelomocytes was performed using TRI REAGENT^®^ (Sigma Aldrich, St. Louis, MO, USA), following the manufacturer’s instructions for DNA isolation. The quality and quantity of DNA were determined on a NanoDrop™ 2000 spectrophotometer (Thermo Fisher Scientific, Waltham, MA, USA). DNA was diluted to a concentration of 12.5 ng/µL using DMPC water. Then, 2 µL of diluted DNA solution was mixed with 2 µL 2 × DNA denaturation buffer (200 mM NaOH, 20 mM EDTA) and incubated at 95 °C for 10 min. Samples were placed on ice to cool and then centrifuged briefly. Subsequently, 4 µL 20 × saline sodium citrate buffer (SSC: 3 M NaCl, 0.3 M sodium citrate, pH 7.0) was added to each sample and kept on ice.

Hybond^TM^ P 0.45 µm PVDF membranes (Thermo Fisher Scientific, Waltham, MA, USA) were used for dot blot analysis. Per sample, a minimum of 1 cm^2^ was marked carefully using a sharp pencil and a ruler. The handling of membranes was performed exclusively with forceps. The membrane was activated using 100% methanol for a few seconds and washed with 1 × Tris-buffered saline containing TWEEN^®^ 20 (TBS-T: 137 mM NaCl, 20 mM Tris HCl, pH = 7.6 and 1.23 M Tween^®^ 20 (Carl Roth GmbH & Co. KG, Karlsruhe, Germany)). The membrane was left for 3 min at room temperature placed on TBS-T-soaked blotting paper. Then, 8 µL per sample (25 ng total DNA and buffers) was applied to the designated areas on the membrane. The membrane was left at room temperature until all dots were completely absorbed and then cross-linked with UV light for 3 min using the ChemiDoc™ MP Imaging System (Bio-Rad Laboratories, Hercules, CA, USA). Pure (100%) methanol was used for a few seconds to reactivate the membrane followed by a washing step with TBS-T. For blocking, 10 mL TBS-T including 5% dissolved BSA (Albumin Fraction V, Carl Roth GmbH & Co. KG, Karlsruhe, Germany) was applied for 1 h at room temperature on a shaker. Then, the membrane was incubated with the mouse monoclonal anti-5mC antibody (SAB2702243, Sigma-Aldrich, St. Louis, MO, USA), which was diluted 1:1000 in 10 mL TBS-T with 5% BSA at 4 °C on a shaker for 24 h. The membrane was washed three times with TBS-T for 10 min and incubated with the goat anti-mouse IgG labelled with horseradish peroxidase (A3562, Sigma-Aldrich, St. Louis, MO, USA) (1:10,000 in TBS-T with 5% BSA) for 1 h at room temperature on a shaker followed by three further washing steps using TBS-T for 10 min.

The same procedure was applied for detecting 5hmC, with the following modification: 500 ng of total DNA was used per dot. Blocking and antibody incubation were performed with TBS-T including 5% milk powder. The membrane was incubated with anti-5hmC antibody (THP39770, THP Medical Products, Vienna, Austria) at a concentration of 1:10,000. For detection, the rabbit anti-mouse antibody (ab6421, Abcam, Cambridge, UK) was used at a concentration of 1:10,000.

Detection was conducted using ECL Select™ Western Blotting Detection Reagents (Thermo Fisher Scientific, Waltham, MA, USA) on the ChemiDoc™ MP Imaging System (Bio-Rad Laboratories, Hercules, CA, USA). Subsequently, the 1 mL peroxide solution and 1 mL luminol solution was mixed and applied to the membrane and incubated for 2 min at room temperature. The excessive fluid was removed and chemiluminescence was detected with an exposure time of 0.5 s. Image-Lab 4.1 (Bio-Rad Laboratories, Hercules, CA, USA) was applied to quantify the dots relative to the controls, and the background-adjusted volume was used for signal detection. Analysis settings were the local background subtraction method for volume and the linear quantity regression method for the analysis.

### 2.6. Statistical Analysis

Statistical analyses was performed with GraphPad Prism 8.3 software (GraphPad Software, San Diego, CA, USA). Outliers were calculated using the Grubbs test and were considered significant at *p* < 0.01. Outliers were excluded from further analysis. Normality distributions were calculated using the Kolmogorov–Smirnov test if possible. Otherwise, the Shapiro–Wilk test was used to identify data distribution. Normally distributed data were analyzed by performing Student’s *t*-tests. If f-tests revealed significant differences in variance (*p* < 0.05) Welch’s correction was performed. Non-normally distributed data were analyzed using the Mann–Whitney U test.

Multidimensional scaling analysis (MDS) and correlation tests were performed using RStudio Version 1.3.1073 [51]. MDS was performed using the script “cmdscale”. The analyzed variables of tissue and coelomocyte samples were DNMT1, TET, MT2 and 5mC. The number of samples used for the analysis was as follows: C = 5 and 7; A = 6 and 5; Cd = 5 and 8; CdA = 3 and 13 for coelomocytes and tissue, respectively. The script “ordiellipse” (package “vegan” [52]) was used to generate an ellipse around treatment groups (confidence of 90%). ANOVA and Tukey’s HSD tests on MDS score values (*p* < 0.05) revealed a significant difference between treatment groups in the first two dimensions.

## 3. Results

### 3.1. Quantification of Gene Expression

In general, across all treatments, gene expression levels of MT2, DNMT1 and TET were significantly higher in coelomocytes compared with tissue samples. Significance levels are presented in Table 1.

**Figure 1:**
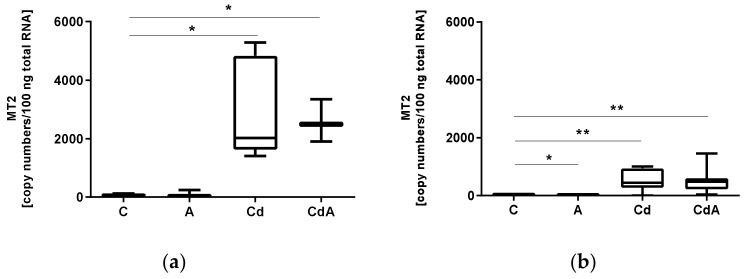
(**a**) mRNA copy numbers of Metallothionein 2 (MT2) in *L. terrestris* coelomocytes and (**b**) tissue of control (C) and exposed individuals (5-aza-2′-deoxycytidine (A); 200 mg/kg CdCl_2_ (Cd); 5-aza-2′-deoxycytidine and 200 mg/kg CdCl_2_ (CdA)). Stars indicate significant differences between treatments and the control (*p* < 0.05 (*); *p* < 0.01 (**)). Replicates per treatment analyzed in coelomocytes: C = 5, A = 6, Cd = 5, CdA = 3. Replicates per treatment analyzed in tissue: C = 7, A = 5, Cd = 8, CdA = 12.

In coelomocytes, MT2 gene expression was significantly increased after 48 h of exposure in Cd- (Welch’s test, *p* < 0.05) and CdA-treated groups (Welch’s test, *p* < 0.05) in comparison to the control (Figure 1A). For tissue, MT2 expression levels significantly increased in Cd (Welch’s test, *p* < 0.01) and CdA (Welch’s test, *p* < 0.01) exposed groups in comparison to the control, whereas the treatment with Aza inhibited MT2 transcription (Mann–Whitney U test, *p* < 0.05) (Figure 1B).

**Figure 2:**
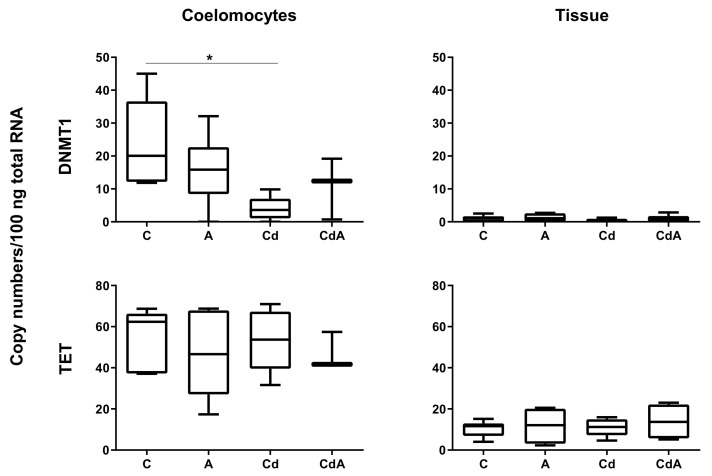
mRNA copy numbers of DNA-methyltransferase 1 (DNMT1) and ten–eleven translocation (TET) in *L. terrestris* coelomocytes and tissue of control (C) and exposed individuals (5-aza-2′-deoxycytidine (A); 200 mg/kg CdCl_2_ (Cd); 5-aza-2′-deoxycytidine and 200 mg/kg CdCl_2_ (CdA)). Stars indicate significant differences between treatments and control (*p* < 0.05 (*)). Replicates per treatment analyzed in coelomocytes: C = 5, A = 6, Cd = 5, CdA = 3. Replicates per treatment analyzed in tissue: C = 6, A = 4, Cd = 6, CdA = 6.

DNMT1 gene expression significantly decreased in coelomocytes in the Cd-exposed group in comparison to the control (Welch’s test, *p* < 0.05). Neither for tissue, nor for coelomocytes, were significant differences in TET gene activity observed (Figure 2). Gene expression levels of DNMT3 showed very low constitutive transcription rates for both tissue and coelomocytes (data not shown).

### 3.2. Global Methylation Level and DNMT Activity

**Figure 3:**
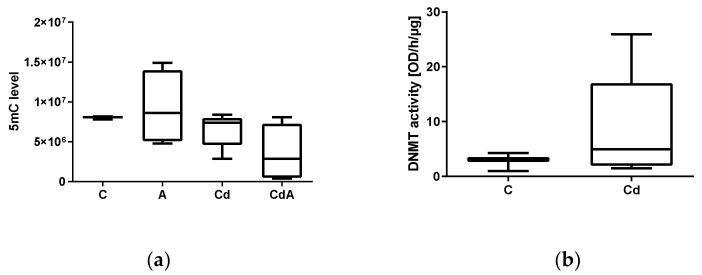
(**a**) Global DNA methylation levels in coelomocytes extruded from control (C, *n* = 4) and exposed *L. terrestris* specimens (5-aza-2′-deoxycytidine (A), *n* = 4; 200 mg/kg Cd (Cd) *n* = 5; 5-aza-2′-deoxycytidine and 200 mg/kg CdCl_2_ (CdA), *n* = 4) after 48 h of exposure. (**b**) Total DNMT protein activity (DNMT1 and DNMT3) of coelomocytes extruded from control (C, *n* = 3) and Cd-exposed (200 mg/kg CdCl_2_ (Cd, *n* = 6)) *L. terrestris* individuals after 48 h of exposure.

Global DNA methylation levels of cytosines in tissue samples did not differ significantly (Supplementary Figure S1A). In coelomocytes, a slight, but not significant decrease was detected in the CdA-treated group in comparison to the control (Figure 3a). DNMT activity of earthworm coelomocytes did not significantly differ between the control and Cd-treated group (Figure 3b).

### 3.3. Global 5hmc

Cd had no significant influence on global 5hmC levels in DNA extracted from coelomocytes (Supplementary Figure S1B).

### 3.4. Multidimensional Scaling

**Figure 4:**
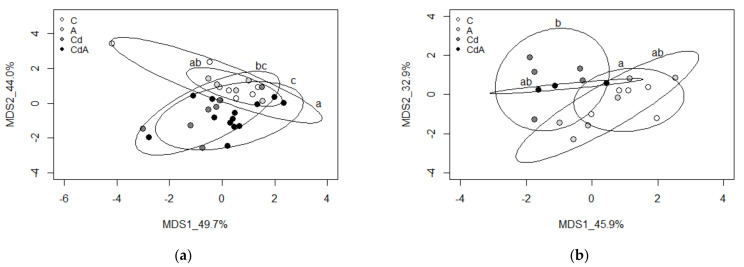
(**a**) MDS analysis using tissue samples and variables DNMT1, TET, MT2 and 5mC. Significance in group separation is based on the MDS2 dimension (shown by letters). The ellipse around the treatment groups indicates a confidence of 90%. (**b**) MDS analysis using coelomocyte samples and variables DNMT1, TET, MT2 and 5mC. Significance in group separation is based on the MDS1 dimension (shown by letters). The ellipse around treatment groups indicates a confidence of 90%.

For tissue samples, MDS analysis revealed significant differences between groups in the second dimension. Cd and CdA groups were significantly different from C (*p* = 0.0043241 and 0.0006196, respectively) and CdA was significantly different from A (*p* = 0.0152221) (Figure 4a).

For coelomocyte samples, MDS revealed a significant difference between C and Cd groups in the first dimension (*p* = 0.0127395) (Figure 4b).

## 4. Discussion

We tested the effects of the heavy metal Cd and the demethylation agent Aza in the earthworm *Lumbricus terrestris* with a special focus on MT2-related Cd detoxification, changes in global 5mC and 5hmC levels, and the activity of DNA methylation-associated genes in coelomocytes and tissue samples.

Multidimensional scaling clearly revealed similar separation between treatment groups for both tissue and coelomocyte samples. For tissue samples, Cd and CdA showed difference from the control group; however, because Aza treatment did not differ from the control, we conclude that the effects were Cd-related. A similar trend was evident for coelomocyte samples; however, only the Cd group differed significantly from the control.

Compared with tissue samples, gene expression in coelomocytes was higher across all treatments. Increased MT2 levels in coelomocytes might be due to their specialized function in Cd detoxification [45]. DNMT tissue-specific expression and altered expression in response to different types of stress has been demonstrated previously [53]. Another study on DNA methylation genes revealed that DNMT as well as TET are expressed differentially in tissues and during development [54]. Herein, we confirmed a tissue-specific expression of DNMT1 as well as TET in earthworms and propose that DNA methylation takes over specialized functions in coelomocytes.

Astonishingly Aza inhibited MT2 gene expression, which was not observed in the presence of Cd, indicating that Aza affects basal gene activity, but not Cd-inducible MT2 transcription in *L. terrestris* tissue. Interestingly, the opposite effect was described in vertebrates. In rat liver cells, Aza treatment led to a dose-dependent increase in MT expression and to hypomethylation of the MT gene, which was suggested to play a role in enhanced expression rates [55].

Aza inhibits DNMT1 and requires the cells to divide in order to demethylate their genes [37]. In our study, Aza treatment did not influence global DNA methylation levels. Therefore, either the used concentrations were too low, Aza could not bind to *L. terrestris* DNMT1 protein, or the treatment time was too short for Aza to take effect. Aza treatment in invertebrate species showed different effects on DNA methylation patterns. One study investigated the effects of an endoparasitoid wasp on DNA methylation patterns and altered gene expression in its host species, the moth *Plutella xylostella.* As part of this study, Aza was injected into moth larvae, leading to a dose-dependent decrease in 5mC intensity 48 h post-injection [33]. In 2–3-day-old silkworm *Bombyx mori* pupa, Aza led to a decrease in DNA methylation at specific CpG sites in wing-specific genes [34]. In callow *Bombus terrestris* bumblebee workers, Aza treatment led to changes in DNA methylation patterns, but the treatment had no effect on adult workers. Regarding DNA methylation pattern changes, Aza treatment induced hypo- as well as hypermethylation changes in several of the observed loci in callow workers. The authors hypothesized that a weak proliferation rate in adult bees could explain their unchanged methylation pattern, because Aza requires the cells to divide in order to fulfill its potential as a demethylating agent [56]. We also performed our experiment in adults and observed that Aza did not demethylate DNA on a global level. Weak proliferation rates could explain unchanged DNA methylation patterns, especially in earthworm coelomocytes, which are known to have weak proliferation rates, as previously shown in *Dendrobaena veneta* [57]. In adult female *Nasonia vitripennis* wasps, Aza treatment led to both hypo- and hypermethylation and strongly depended on the exposure period, tissue type and collection time [58]. Recently, a study in the marine annelid *Platynereis dumerilii* showed that treatment with 50 µM AZA 24–72 h post-fertilization led to a decrease in global DNA methylation, morphological deformations and increased mortality. All treated larvae died within 10 days post fertilization [35]. Since 48 h of 50 µM Aza treatment led to 100% mortality in earthworms, we used 10 µM Aza for earthworm exposures.

In murine HT22 cells, Aza treatment caused alterations of the cell cycle leading to S-phase arrest, and low doses of Aza significantly reduced DNMT1 gene and protein expression as well as DNMT1 protein activity. Interestingly, this decrease was not observed for total DNMT activity when DNMT3 proteins were included [59]. In our study, Aza had no effect on DNMT1 gene expression, but an Aza-mediated S-phase arrest could explain unchanged global DNA methylation patterns in *L. terrestris*. For a better application of demethylation agents in experimental biology, further studies that test the demethylation potential of several reagents in invertebrate species are needed.

In a recently published study on earthworms exposed to environmentally relevant Cd concentrations, changes in global DNA methylation were observed after 2 weeks, and the authors did not observe differences in gene expression for DNMT1, DNMT3 and TET genes at all examined time points [46]. Compared with the latter, in the present study, higher doses of Cd were used, but still no effect on the activity of the TET gene and global 5hmC levels were observed, indicating that TET protein activity was not affected. We observed very low constitutive transcription rates of DNMT3 in tissue and coelomocytes. Thus, even higher doses of Cd did not show an effect on TET and DNMT3 gene activity. The decrease in DNMT1 gene expression in coelomocytes had no effect on DNMT activity, which could be a reason for unchanged DNA methylation. DNMT1 is known to be post-translationally modified. Therefore, we assumed that a down-regulation of DNMT1 gene expression in earthworms would not necessarily and immediately lead to a change in protein activity. It has further been shown that DNMT gene expression does not reflect global methylation levels [60,61].

Impaired upstream signaling could be a possible explanation for the Cd-induced repressed transcription of DNMT1. Zn and Cd are chemically and physically similar, enabling substitution of these elements, which is known to destabilize proteins containing zinc finger domains [62,63], for example, and consequently affecting the DNA binding affinity [64]. Zinc finger proteins such as GLI1, SP1, SP3, and ZCCHC6 have previously been shown to be involved in DNMT gene regulation [65,66,67,68]. Cd-incorporated Zn finger proteins could explain the toxic effects of Cd, leading to the decrease in DNMT1 gene activity.

Earthworms were shown to develop an acclimation mechanism when exposed to low doses of Cd; Cd exposure leads to global DNA hypermethylation [44]. We hypothesized that these Cd-induced epigenetic changes could explain the enhanced MT2-related detoxification mechanisms, but in *L. terrestris*, MT2 gene expression could not be correlated to changes in MT2 gene body methylation patterns [46]. In the present work, we observed significantly upregulated MT2 transcription in Cd-exposed groups in tissue and coelomocytes. No changes in global DNA methylation levels occurred; therefore, we suggest that Cd detoxification is not related to global DNA methylation changes, at least not regarding the methylation of cytosines.

We know that in *L. terrestris* exposed to low doses of Cd, hypermethylation is observed after 4 weeks of exposure in coelomocytes and after 2 weeks in tissue [44,46]. Interestingly, high doses of Cd did not change global DNA methylation in our study, neither for coelomocytes nor for tissue after 48 h. In the brine shrimp *Artemia franciscana*, global DNA methylation did not change after two days of Cd exposure [69]. Thus, a change in Cd-induced global DNA methylation strongly depends on the dose and exposure time.

## 5. Conclusions

The results of this study indicate that in earthworm coelomocytes, high doses of Cd repress the gene expression of DNMT1, which is responsible for maintaining DNA methylation. However, downregulated DNMT1 had no effect on total DNMT protein activity and on global DNA methylation levels. Interestingly, Aza also had no effect on global methylation, but inhibited basal MT2 gene expression in *L. terrestris* tissue. However, MT2 transcription was significantly upregulated in Cd- and CdA-treated groups, meaning that DNA methylation changes and Aza treatment do not affect the induction MT2.

## Figures and Tables

**Table 1:** *p*-values and statistical tests applied to identify differences in gene activity within treatment groups between coelomocytes and tissue.

Gene	Treatment	Statistical Test	*p* Value
MT2	C	Student’s *t*-test	0.0117
	A	Mann–Whitney U test	0.0606
	Cd	Welch’s test	0.0324
	CdA	Student’s *t*-test	<0.0001
DNMT1	C	Welch’s test	0.0216
	A	Welch’s test	0.019
	Cd	Mann–Whitney U test	0.0455
	CdA	Welch’s test	0.2101
TET	C	Welch’s test	0.0025
	A	Student’s *t*-test	0.0208
	Cd	Welch’s test	0.0025
	CdA	Student’s *t*-test	0.0008

## Data Availability

Data is contained within the article or supplementary material.

## Supplementary Materials

The following supporting information can be downloaded at: https://www.mdpi.com/article/10.3390/toxics10020100/s1, Figure S1: Global 5mC in tissue and 5hmC in coelomocytes.
