# Supplementary material for: DNA Methylation and Detoxification in the Earthworm Lumbricus terrestris Exposed to Cadmium and the DNA Demethylation Agent 5-aza-2′-deoxycytidine
Source: Toxics. 2022 Feb 21;10(2):100. doi: 10.3390/toxics10020100 (PMC8879108; doi:10.3390/toxics10020100)
Supplement: Supplementary file 1 [file toxics-10-00100-s001.zip › toxics-1551281-supplementary.pdf]

Supplementary material: DNA methylation and detoxification in the earthworm *Lumbricus terrestris* exposed to cadmium and the DNA demethylation agent 5-aza-2′-deoxycytidine

Gerhard P. Aigner, Pamela Nenning, Birgit Fiechtner, Maja Šrut, Martina Höckner

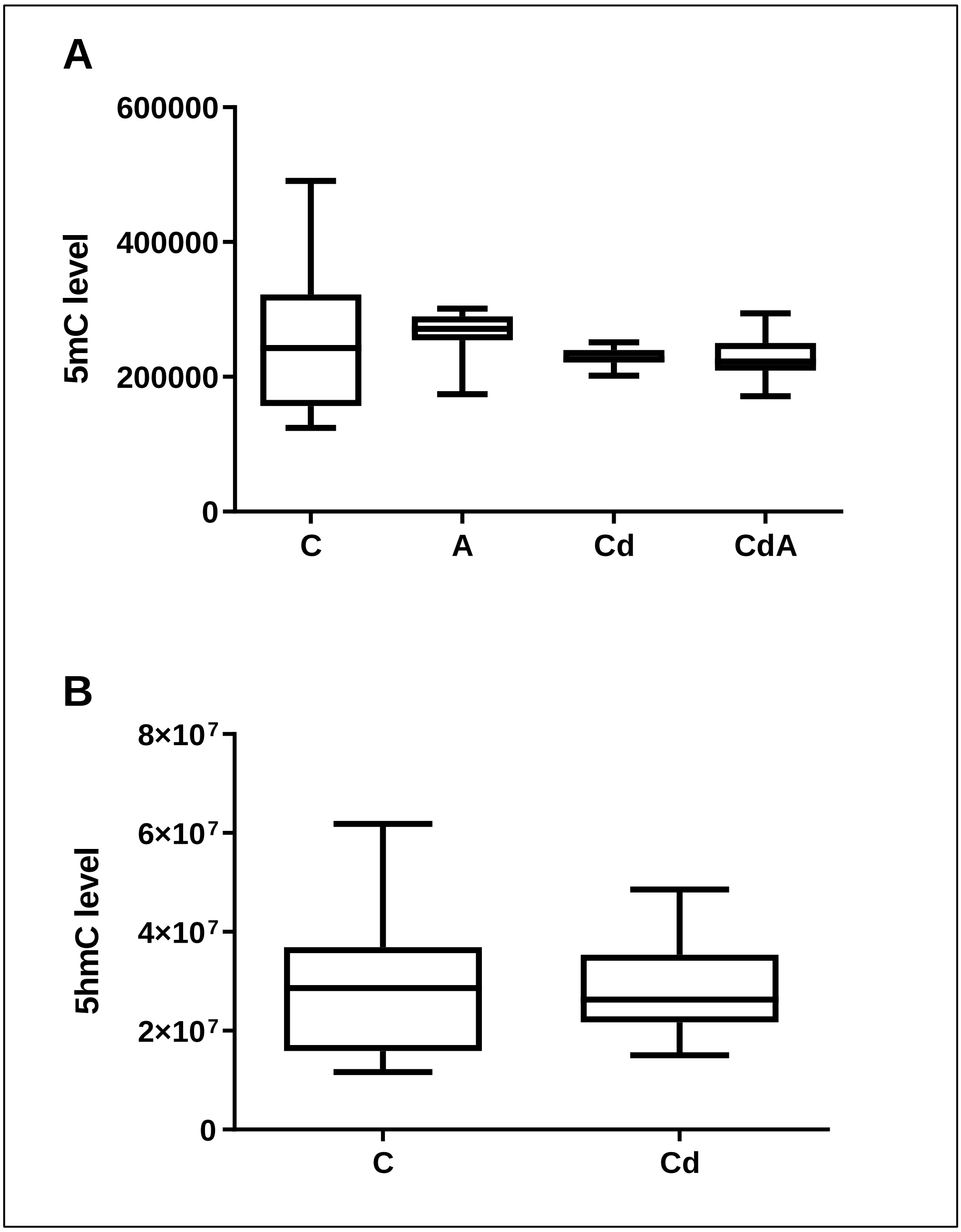

Figure S1. Global DNA methylation levels in tissue from control (C, n = 6) and exposed *L. terrestris* individuals (5-aza-2′-deoxycytidine (A), n = 7; 200 mg/kg Cd (Cd), n = 7; 5-aza-2′-deoxycytidine and 200 mg/kg Cd (CdA), n = 13) after 48 h of exposure (**A**). Global levels of hydroxymethylcytosine in coelomocytes extruded from control (C, n = 10) and Cd-exposed *L. terrestris* individuals (200 mg/kg Cd (Cd), n = 10) after 48 h of exposure (**B**).
